# Global temperature anomaly prediction by using additive twin LSTM networks

**DOI:** 10.1038/s41598-026-37255-x

**Published:** 2026-01-28

**Authors:** Cemal Keles, Burhan Baran, Baris Baykant Alagoz

**Affiliations:** 1 Department of Electrical and Electronics Engineering, Inonu University, Malatya, Türkiye; 2 Ministry of Environment, Urbanisation and Climate Change, Malatya, Türkiye; 3 Department of Computer Engineering, Inonu University, Malatya, Türkiye

**Keywords:** Climate change, Global warming, Temperature anomaly, Forecasting, Climate sciences, Environmental sciences, Mathematics and computing

## Abstract

Due to the complexity of climate systems, data-driven modeling based on observed time series data is essential for predicting future climatic trends. This study aims to improve the long-term global temperature anomaly forecast performance of Long Short-Term Memory (LSTM) based neural network models. Although several LSTM variants and hybrid architectures have been suggested for time series data prediction problems, the long-term forecast performance of these models may not be satisfactory in practice. To address solution of these problems, firstly, authors focused on evaluating the forecast performance of models and suggested performance and test assessment procedures. Secondly, authors suggest an Additive Twin LSTM (AT-LSTM) model that can improve the forecast performance for the global temperature anomaly. Our test on the Berkeley Global Temperature Anomaly dataset demonstrates that the proposed AT-LSTM can improve performance relative to conventional LSTM variants in long-term forecasting. Authors observed that global temperature trend projections of the AT-LSTM models for 20 years in future are consistent with expectations of climate organizations and projections in other works. The AT-LSTM models forecasted an average of 1.415 °C with ± 0.073 °C error in the year 2042 and this indicates the strong potential of major climate changes in the near future of Earth.

**Supplementary Information:**

The online version contains supplementary material available at 10.1038/s41598-026-37255-x.

## Introduction

Global warming is one of the global-scale climatic transition problems, leading to increases in global temperatures, glaciers melting, sea-level rise, expanding desert zones, and abnormal climatic changes such as intermittent hurricanes, floats and landslides^1^. Among these effects, the mean global temperature rises have a significant impact on daily-life activities from energy consumption, agricultural and livestock activities, as well as, its serious depression on social, economic and environmental systems^2^. Therefore, forecasting global temperature anomalies is crucial for improving resource management policies, understanding long-term climate change trends, and taking measures to prevent or mitigate associated problems^3,4^. To address this problem, time series prediction is one of the developing fields of big data analysis^5^. Traditional methods for time series data forecasting have not been successful in capturing complicated sequential relationships and long-term dependencies^6^.

Recurrent neural networks (RNN) can inherently learn sequential relations in time series data. Popularly, long short-term memory (LSTM) models have become widely used due to their ability to manage both short-term and long-term dependencies in time series data patterns compared to conventional RNNs^3^. In other words, the learning process of LSTM models is assumed to be relatively insensitive to the sequence length that is important in case of long-term dependencies in datasets^7^. This property of LSTM is very significant for the data-driven modeling practice because deciding the necessary sequence length of RNNs for a real-world data is not an easy problem. To possess this useful property, LSTMs involve specially designed building blocks: A classical LSTM unit is composed of a cell state and three gates, which are known as an input gate, an output gate^8^, and a forget gate^9^. Cell states mainly function as selective memories and gate performs for updating and forgetting information. Following the success of LSTMs in practice, a gated type of RNNs, known as the gated recurrent unit (GRU), was suggested to achieve similar capabilities with fewer parameters. The GRU has similar elements to those of LSTMs. Specifically, the GRU has a two-gate system (i.e., update and reset gates) to handle long-term temporal dependencies in time-series prediction^7^. Practically, GRUs are simpler and faster than LSTMs because they lack a separate cell state^10^. LSTMs are preferable for data sequences that have long-term dependencies, whereas GRU can be potentially effective in learning the relations that do not involve the long-term dependencies. Recent studies have also shown that hybrid architectures combining LSTM, CNN, and GRU models can enhance predictive performance depending on the characteristics of the dataset^11^. Table 1 briefly summarizes research works in the literature, which are related to temperature prediction. Changes in global temperature anomaly were analyzed by using the LSTM, and the NOAA and NASA datasets were used for training LSTM models^12^.

To improve consistency in the collected sequential temperature anomaly data, we used the Berkeley Global Temperature Anomaly monthly data averaged over five years. The five-year long averaging monthly data can significantly reduce uncertainty in global temperature anomaly observations. In our numerical experiments, we observed that the long-term global temperature forecasting performance of LSTM models and many hybrid forms may not be dependable. Even though they can be highly accurate in the short-term prediction (in the next temperature prediction), recursive prediction efforts for long-term temperature forecasting towards future in time, which is called future forecasting, cannot be consistent enough. To address this limitation, we propose two twin-branch architectures: the Additive Twin LSTM (AT-LSTM) and the Multiplicative Twin LSTM (MT-LSTM). The proposed AT-LSTM differs fundamentally from conventional LSTM and GRU architectures by employing a twin-branch recurrent design that allows the model to learn partially uncoupled dominant dynamics within the sequence. This structural distinction enhances long-horizon forecasting stability, as confirmed by benchmark and climate-data evaluations, where AT-LSTM can improve performance relative to representative recurrent models in RMSE, MAE, and R^2^ metrics. Our results indicate that the AT-LSTM model improves future forecasting performance for global temperature anomaly data compared to conventional LSTM and hybrid LSTM architectures. Main advantageous comes from the twin LSTM utilization with a common decoder network, where each LSTM can focus on learning behavior of an uncoupled dominating climate dynamic.

**Table 1:** A brief survey for temperature prediction models.

Works	Methods	Predicted data	Performance/remarks
Diffenbaugh et al. (2023)^13^	ANN, XAI	Global warming	1.5 °C global warming threshold is expected to be reached in 2033–2035
Guo et al. (2024)^14^	ANN, RNN, LSTM, CNN, CNN-LSTM	Monthly climate parameter	CNN-LSTM model provides higher accuracy with MAE, RMSE, R²
Uluocak et al. (2024)^15^	LSTM-CNN, GRU-CNN	Daily air temperature	LSTM-CNN and GRU-CNN models make higher accuracy predictions with MAE, RMSE, NSE, and R²
Guo et al. (2023)^16^	ANN, GRU, LSTM, CNN, CNN-GRU, CNN-LSTM	Atmospheric temperature	They use R², RMSE, MAE as success scales. The best R² value is 0.9952 with ANN. Average atmospheric temperature in 2030 is predicted to be 17.23 °C
Hamdan et al. (2023)^12^	A mathematical model with RNN and LSTM	Global temperature and greenhouse gas emission changing	LSTM model obtains high accuracy results with RMSE: 2.018 (NOAA) and 0.814 (NASA)
Li et al. (2023)^17^	SARIMA-LSTM	Air temperature	The accuracy of the model increase from 10.0% to 27.7%
Hou et al. (2022)^18^	CNN-LSTM	Hourly air temperature	R²: CNN-LSTM is 0.7258, LSTM is 0.5949, CNN is 0.5291
Haque et al. (2021)^19^	SRN, GRU, LSTM, CNN, CNN-LSTM, GRU-LSTM	Hourly air temperature	The lowest RMSE (1.691 °C) is obtained with GRU-LSTM
Zhao et al. (2021)^20^	CNN-GRU-RPASM	Air temperature	CNN-GRU-RPASM shows the best performance compared to traditional methods
Zhang et al. (2020)^21^	CRNN (model consisting of CNN and RNN components)	Air temperature	For the CRNN, MAE is calculated as 0.907 °C and RMSE is as 1.697 °C.
Gong et al. (2024)^22^	CNN-LSTM	Daily average temperature	The predicted curve shows strong agreement with the actual test data
Li et al. (2024)^1^	CNN-LSTM	Temperature	obtained MSE (3.26217) and RMSE (1.80615) values are higher than the traditional methods
Karabulut et al. (2022)^4^	LSTM	Air temperature	R^2^ value is 0.9937 for LSTM, 0.8869 for SVM

##  Additive twin LSTM (AT-LSTM) for time series data prediction

### Basics of LSTM model

**Fig. 1:**
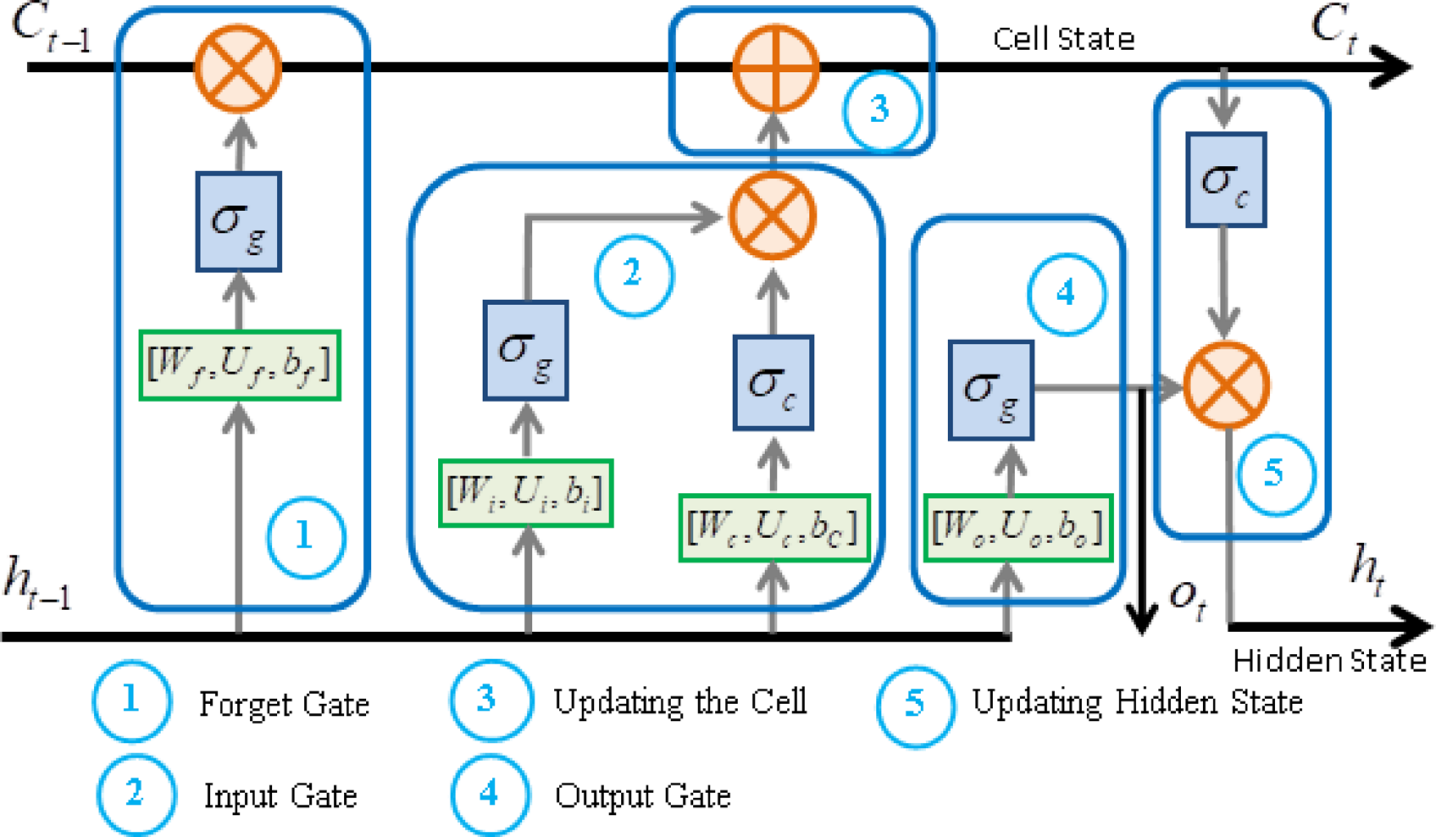
Fundamental elements of a LSTM unit and their mathematical relations.

In principle, it employs information-flow controlling mechanisms (gates) in order to exhibit the long-term memory effect by using short-term memory elements. A basic LSTM unit consists of a cell and three gates that are known as an input gate, an output gate^8^, and a forget gate^9^. Cell states mainly function as selective memories that can convey useful (relevant) information over longer time intervals by means of updating and forgetting control of the gates. Here, the forget gate is designed to forget (suppress) irrelevant information, and the input gate is proposed to update the cell memory with relevant new information. Thus, the cell determines cell states ($\:{C}_{t}$) to maintain useful information over arbitrary time intervals, thereby leading to a long-term memory effect. The output gate determines how the memory is translated at the output at each step. It also contributes to determining the hidden state ($\:{h}_{t}$) by regulating the cell state. This selective memory property makes the LSTM more efficient and flexible in learning which parts of the sequence are important and should be preserved through the information-flow. Figure 1 shows the fundamental elements of a LSTM unit that describes mathematical relations between the basic elements in a LSTM unit. The information in the cell state flows throughout a sequence of subsequent LSTM units, and such unit array forms a long memory effect that is based on selective learned control of short-memory elements in each LSTM unit.

The mathematical foundation of each element can be summarized as:

1. A forget gate determines the amount of information from the previous cell state should be discarded. It calculates the weighted sum of the previous hidden state ($\:{h}_{t-1}$) and the current input ($\:{x}_{t}$), then applies a sigmoid activation function ($\:{\sigma\:}_{g}$) to regulate the gate output between 0 and 1. A value of 0 means “completely forget”, and a value of 1 means “completely keep”.
  1 $$\:{f}_{t}={\sigma\:}_{g}({W}_{f}{x}_{t}+{U}_{f}{h}_{t-1}+{b}_{f})$$

where the weight coefficients in forget gate are denoted by the vectors $\:{W}_{f}$ for the current input $\:{x}_{t}$ and $\:{U}_{f}$ for the hidden state $\:{h}_{t-1}$. They are optimized during the training stage of LSTM via the backpropagation algorithm. This allows learning irrelevant information patterns that should be suppressed through cell states.

2) An input gate determines the amount of new information that will be added to the cell state. It uses two networks:

The first one is a layer neuron network with sigmoid activation that regulates the amount of new information ($\:{\stackrel{\sim}{C}}_{t}$).

2 $$\:{i}_{t}={\sigma\:}_{g}({W}_{i}{x}_{t}+{U}_{i}{h}_{t-1}+{b}_{i})$$

where vectors $\:{W}_{i}$ and $\:{U}_{i}$ stand for weight coefficients for the current input $\:{x}_{t}$ and previous hidden state $\:{h}_{t-1}$, respectively. The $\:{i}_{t}$ takes a value between 0 and 1 and for regulating $\:{\stackrel{\sim}{C}}_{t}$.

The second one is a neuron network with a tanh activation ($\:{\sigma\:}_{c}$) that generates a vector of new candidate values ($\:{\stackrel{\sim}{C}}_{t}$) that is added to the cell state after regulating by the $\:{i}_{t}$.

3 $$\:{\stackrel{\sim}{C}}_{t}={\sigma\:}_{c}({W}_{c}{x}_{t}+{U}_{c}{h}_{t-1}+{b}_{C})$$

The input gate and the candidate cell state are then used to update the cell state. The vectors $\:{W}_{c}$ and $\:{U}_{c}$ are weights for the current input and previous hidden states, respectively. Values of these vectors are optimized during the training stage.

3) A current cell state is updated by weighted sum of the previous cell state $\:{C}_{t-1}$ and the new cell state $\:{\stackrel{\sim}{C}}_{t}$ as follows
  4 $$\:{C}_{t}={f}_{t}\otimes\:{C}_{t-1}+{i}_{t}\otimes\:{\stackrel{\sim}{C}}_{t}$$

Here, $\:{f}_{t}\in\:\left[\mathrm{0,1}\right]$ performs for suppression of the previous cell state $\:{C}_{t-1}$ and $\:{i}_{t}\in\:\left[\mathrm{0,1}\right]$ performs for update with the new cell state $\:{\stackrel{\sim}{C}}_{t}$. The operator $\:\otimes\:$ represents Hadamard product (element-wise product).

4) The output gate decides the next hidden state that is used in the next LSTM unit, and it is also used as the output of the LSTM at each time step. The output state is calculated as
  5 $$\:{o}_{t}={\sigma\:}_{g}({W}_{o}{x}_{t}+{U}_{o}{h}_{t-1}+{b}_{o})$$

where the $\:{W}_{o}$ and $\:{U}_{o}$ denotes the weight coefficient vectors for optimizing during the training stage. The terms $\:{U}_{o}{h}_{t-1}$ and $\:{U}_{c}{h}_{t-1}$ stand for the learned dynamics response from the data sequence and they are essential components in determination of the next hidden state in from of

6 $$\:{h}_{t}={O}_{t}\otimes\:{\sigma\:}_{c}\left({C}_{t}\right)$$

### Additive twin LSTM

In nature, data sequences, which are collected from complex systems, can involve more than one uncoupled dominating dynamics, which can be almost independently acting^23–25^, and measured data involves components from these dynamics. Inherently, a composition of these dominating dynamic factors can establish the sequential relations in the collected data sequences. For the case of uncoupled two dominating dynamics, state transitions can be expressed $\:{d}_{t+\mathrm{1,1}}={F}_{1}\left({d}_{t,1},{x}_{t,1}\right)$ and $\:{d}_{t+\mathrm{1,2}}={F}_{2}\left({d}_{t,2},{x}_{t,2}\right)$, the measured data sequence can be expressed in the form of

7 $$\:{y}_{t}=G({d}_{t,1},{d}_{t,2})$$

where the function $\:G(.)$ represents the measurement function, which can be referred to as the decoding function. In multi-component systems, the measurement function (decoding function) can be expressed as a weighted sum of each dynamic factors. (See the property of approximation to independent recurrent relations in Supplementary Material). For data-driven modelling of complex systems with uncoupled multi-dynamic factors, the multi-dynamics modeling approach based on consideration of uncoupled dominating dynamics can present potential of better expressing sequential relations in data sequences. To benefit from this asset, we considered a twin additive LSTM model, where each LSTM can focus on learning one of uncoupled dominating dynamics transition functions ($\:{F}_{1}$,$\:\:{F}_{2}$) and the neural decoding network learns the function of the measurement function ($\:G)$.

In fact, the global temperature anomaly measurements involve impacts of more than one weakly-coupled or uncoupled dynamic factors, such as dynamics associated with global scale CO_2_ emission and Albedo perturbation etc. Energy budget models indicate that global temperature evolution is driven by multiple uncoupled components of the climate system (e.g., radiative forcing response, ocean–atmosphere adjustments, and slow–fast feedback mechanisms)^26–28^. These models suggest that climate dynamics naturally consist of distinct, partially independent temporal processes. This physical characteristic directly aligns with the structural design of the proposed AT-LSTM architecture, in which two parallel recurrent branches are intended to learn disentangled and uncoupled dynamic modes of the underlying system. For these reason, two LSTM approach well suits for the solution of the global temperature anomaly forecasting problem.

**Fig. 2:**
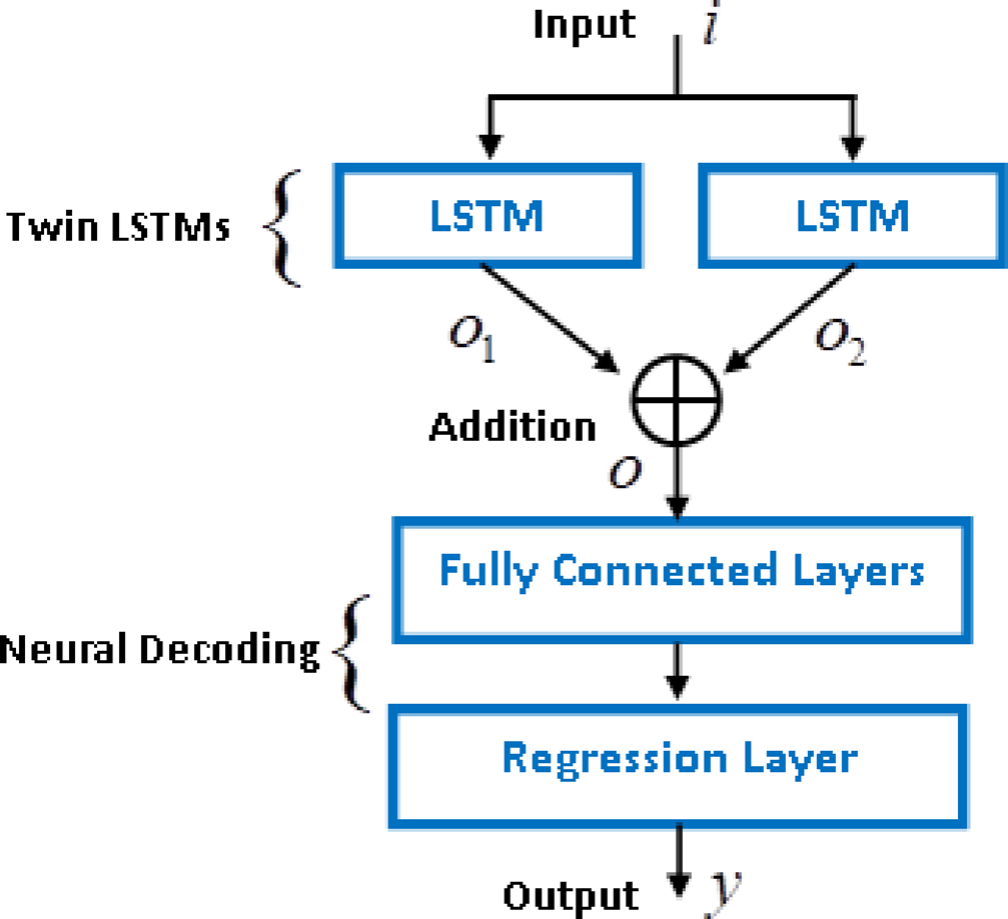
Basic blocks of the AT-LSTM.

In this perspective, the proposed AT-LSTM implements the addition of two identical LSTMs. Figure 2 shows basic blocks of the AT-LSTM that combines two identical LSTM blocks by using an additional block and a neural decoder that yields the output of the model. This implementation provides behavioral and computational benefits: For behavioral benefits, two separate LSTMs can focus on learning two uncoupled dynamics, which are dominative in the composition of sequential relations in the data sequence. For computational benefit, the outputs of two LSTM are added and the sum of two sigmoid functions at the output gates forms a joint activation characteristic. In other words, sum of the two sigmoid functions ($\:{\sigma\:}_{g}\left(v\right)=1/(1+{e}^{-v})$) establishes a activation function that can be expressed in the form of a joint activation as

8 $$\:{\sigma\:}_{sg}({v}_{1},{v}_{2})={\sigma\:}_{g}\left({v}_{1}\right)+{\sigma\:}_{g}\left({v}_{2}\right)=\frac{2+{e}^{-\left({v}_{1}\right)}+{e}^{-\left({v}_{2}\right)}}{(1+{e}^{-\left({v}_{1}\right)})(1+{e}^{-\left({v}_{2}\right)})}$$

By considering the Eq. (8), the joint activation function $\:{\sigma\:}_{sg}({v}_{1},{v}_{2})$ can map output of LSTM networks into the range of 0 to 2 in additive form, and it provides range expansion at input of the neural decoding part.

To form the AT-LSTM network, the outputs of two LSTM networks are summed:

9 $$\begin{gathered} \:o_{t} = o_{{t,1}} + o_{{t,2}} = \sigma \:_{g} \left( {W_{{o,1}} x_{{t,1}} + U_{{o,1}} h_{{t - 1,1}} + b_{{o,1}} } \right) + \sigma \:_{g} \left( {W_{{o,2}} x_{{t,2}} + U_{{o,2}} h_{{t - 1,2}} + b_{{o,2}} } \right) \hfill \\ = \sigma \:_{{sg}} (W_{{o,1}} x_{{t,1}} + U_{{o,1}} h_{{t - 1,1}} + b_{{o,1}} ,\:W_{{o,2}} x_{{t,2}} + U_{{o,2}} h_{{t - 1,2}} + b_{{o,2}} ) \hfill \\ \end{gathered}$$

The output gates can be expressed in the form of additive sigmoid activation as follows

10 $$\:{o}_{t}={o}_{t,1}+{o}_{t,2}=\frac{2+{e}^{-({W}_{o,1}{x}_{t,1}+{U}_{o,1}{h}_{t-\mathrm{1,1}}+{b}_{o,1})}+{e}^{-({W}_{o,2}{x}_{t,2}+{U}_{o,2}{h}_{t-\mathrm{1,2}}+{b}_{o,2})}}{(1+{e}^{-({W}_{o,1}{x}_{t,1}+{U}_{o,1}{h}_{t-\mathrm{1,1}}+{b}_{o,1})})(1+{e}^{-({W}_{o,2}{x}_{t,2}+{U}_{o,2}{h}_{t-\mathrm{1,2}}+{b}_{o,2})})}$$

This section theoretically considers mechanisms that make the AT-LSTM more advantageous in learning sequential relations compared to the conventional LSTM.

### A forecast performance evaluation procedure for time series data

**Fig. 3:**
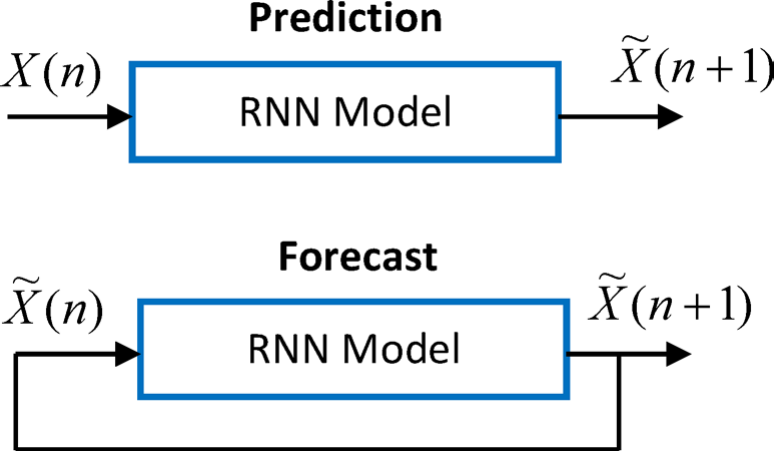
Prediction state (upper figure) and forecasting state (bottom figure).

**Fig. 4:**
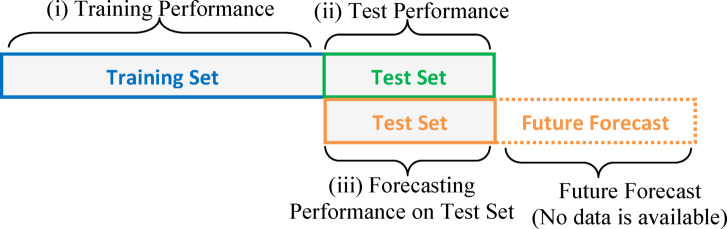
Performance evaluation regions on time-series dataset.

RNNs can learn sequential relations through sequential data. To evaluate RNN model performance, it is useful to distinguish the prediction error, which refers to the error for predicting data instance in the dataset, and the forecast error, which refers to the error for a forecasted sequence that does not exist in the dataset. For prediction performance evaluation, one applies an element of the sequence ($\:X\left(n\right)$) from the dataset and predicts the next element ($\:\stackrel{\sim}{X}(n+1)$) in the dataset. For forecasting performance evaluation, one applies a predicted element ($\:\stackrel{\sim}{X}\left(n\right)$) and predicts the next elements ($\:\stackrel{\sim}{X}(n+1)$). Therefore, it forms feedback from the output of the RNN to the input of RNN and enables consequential future forecasting for a time horizon. However, in this case, this feedback loop causes accumulation of errors in long-term performance evaluation. Figure 3 depicts the prediction and forecast states of RNN models. Consequently, the prediction performance based on training and test data does not truly express the forecast performance of RNN for time series data. In fact, the prediction performance expresses the performance mainly for reproducing the dataset by using elements of the dataset. The forecast performance can express the performance of estimating future elements of a time series. For this reason, the future estimation performance of RNNs should be considered by using the forecast performance. However, assessment of future forecasting performance is a complicated problem because future data is not available, yet. For this case, the nearest forecast performance (the best forecast performance estimate) for the long-term future forecasting can be estimated by considering the forecast performance of the model at the latest part of the dataset. This part can be the last section of test datasets as illustrated in Fig. 4. The test dataset is commonly formed by using the last part (the most recent part) of the time series datasets. Due to being the closest to future prediction, the forecast performance on the test dataset can be used for the nearest forecast performance estimation of future prediction performance of RNNs. Figure 4 describes performance regions on the sequential dataset.

In the long-term forecast process, the forecasted element is used as input in order to predict the next value of the model, and this feedback in the forecasting process causes the accumulation of model prediction errors in the long-term processing. This error accumulation can mislead the forecasting process. Consequently, the forecasting error of RNNs can grow as the forecasting horizon expands into the future, a limitation that has also been emphasized in recent studies on data-driven long-term prediction frameworks^29,30^.

## Global temperature anomaly forecast

### Berkeley’s global temperature anomaly dataset

The temperature anomaly indicates temperature differences in measured temperatures relative to the average reference temperatures of a reference period^31^. It is an important observational parameter for climate change studies. Berkeley Temperature Anomaly (Berkeley Earth Surface Temperature-BEST) data^32^ are generally utilized in global warming and climate change studies^22,33–37^. The BEST has been widely used for monitoring temperature changes on the Earth’s surface^38^. This dataset provides long-term (1850–2024) temperature anomaly from temperature measurements from hundreds of meteorological stations worldwide^32^. It includes temperature anomaly data for monthly, annual, five-year average, ten-year average, and twenty-year averages. Short-term (monthly and even annual) temperature data can usually contain noise and short-term fluctuations. The long-term temperature averages are useful to compensate the effects of measurement noises and short-term fluctuations in the temperature data, and they provide more reliable results that can reveal general climate trends. The analyses of global temperature anomalies should be independent of transient local impacts. Evidently, long-term temperature averages can be more expressive dominating trends in climatic changes, such as signs of the warming or cooling trends. For this reason, five-year average values ​​were considered in this study, and temperature anomaly data between the 6th month of 1852 and the 3rd month of 2022 were used in our numerical study. This time interval excludes the earliest years due to sliding window average operation. This 5-years averaging improves the reliability of the input sequence used for neural network training. The resulting 170-year time series provides a sufficiently large dataset for training deep learning models and for analyzing long-term climate dynamics. The complete dataset is publicly accessible through the Berkeley Earth repository, allowing independent verification and replication of all preprocessing procedures.

### Some benchmark functions for global warming trends

Benchmark functions are commonly preferred to evaluate performance of algorithms (e.g., optimization algorithm, machine learning algorithms) in a controlled test environment (without uncertainty, environmental disturbances, measurement noise etc.). These functions are used to produce synthetically generated data for specific test cases and allows comparison of algorithms for those specific cases. We suggested three different benchmark functions in Table 2. These functions can characteristically simulate extreme temperature anomaly trends and produce synthetic data to benchmark forecast models.

**Table 2:** The suggested benchmark functions and parameters.

Benchmark functions	Equations	Parameters
Exponential function	$\:{y}_{1}={\mathrm{e}}^{a\mathrm{n}}$	$\:a=0.1$
Sinusoidal function with exponential amplitude	$\:{y}_{2}={\mathrm{sin}\left(2\pi\:fn\right)\mathrm{e}}^{a\mathrm{n}}$	$\:f=1$ $\:a=0.1$
Sinusoidal function with exponential offset	$\:{y}_{3}={A\mathrm{sin}\left(2\pi\:fn\right)+\mathrm{e}}^{a\mathrm{n}}$	$\:A=0.1$ $\:f=1$ $\:a=0.1$
Nonstationary trend–sinusoidal function with additive noise	$\:{y}_{4}={n}^{a}+A\mathrm{sin}\left(2\pi\:fn\right)+\epsilon\:$	$\:a=0.05$; $\:A=0.1$; $\:{\upepsilon\:}=0.05\left(rand\right(1,length\left(n\right)-0.5)$

**Fig. 5:**
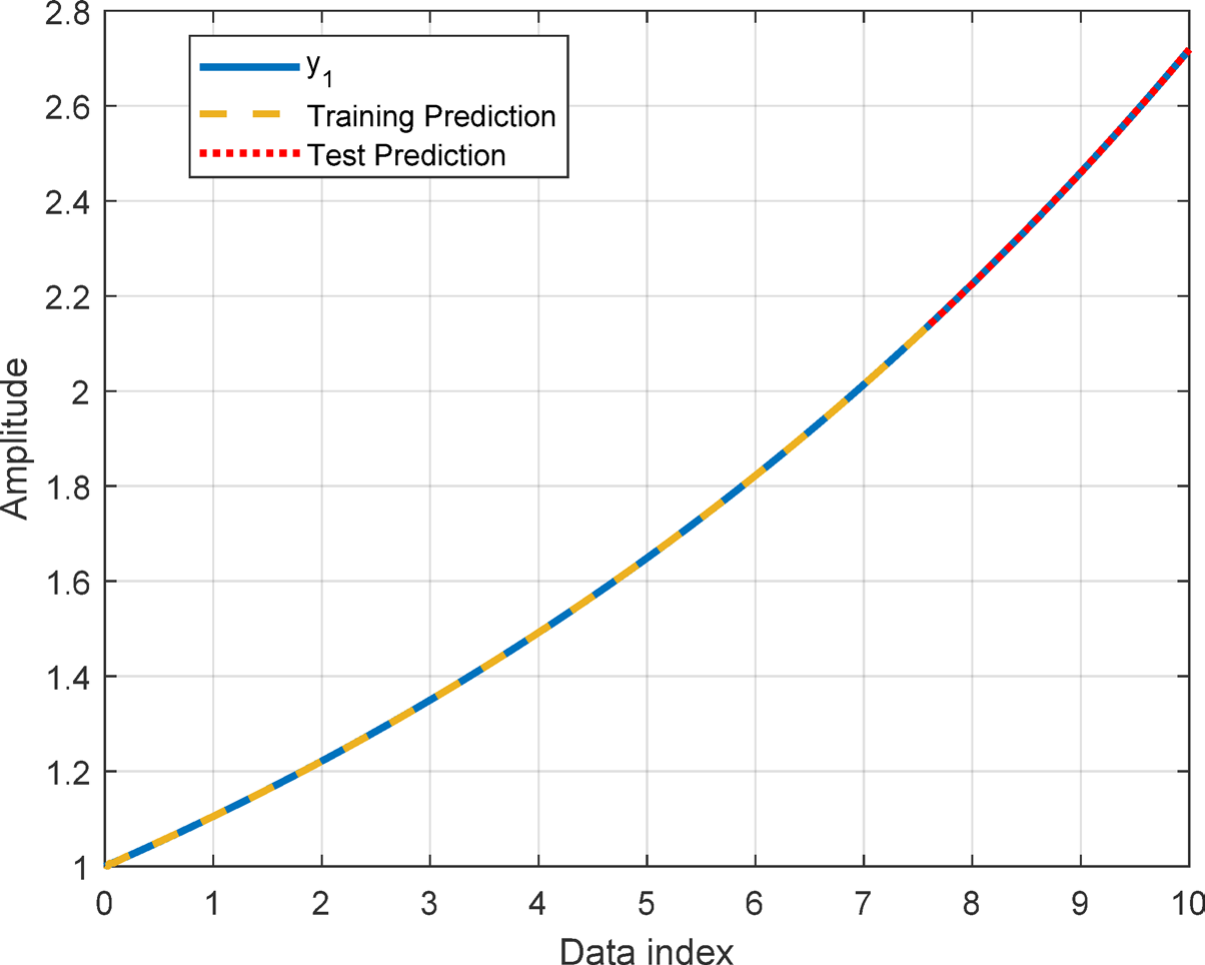
Training and test performances for the benchmark function $\:{y}_{1}$.

**Fig. 6:**
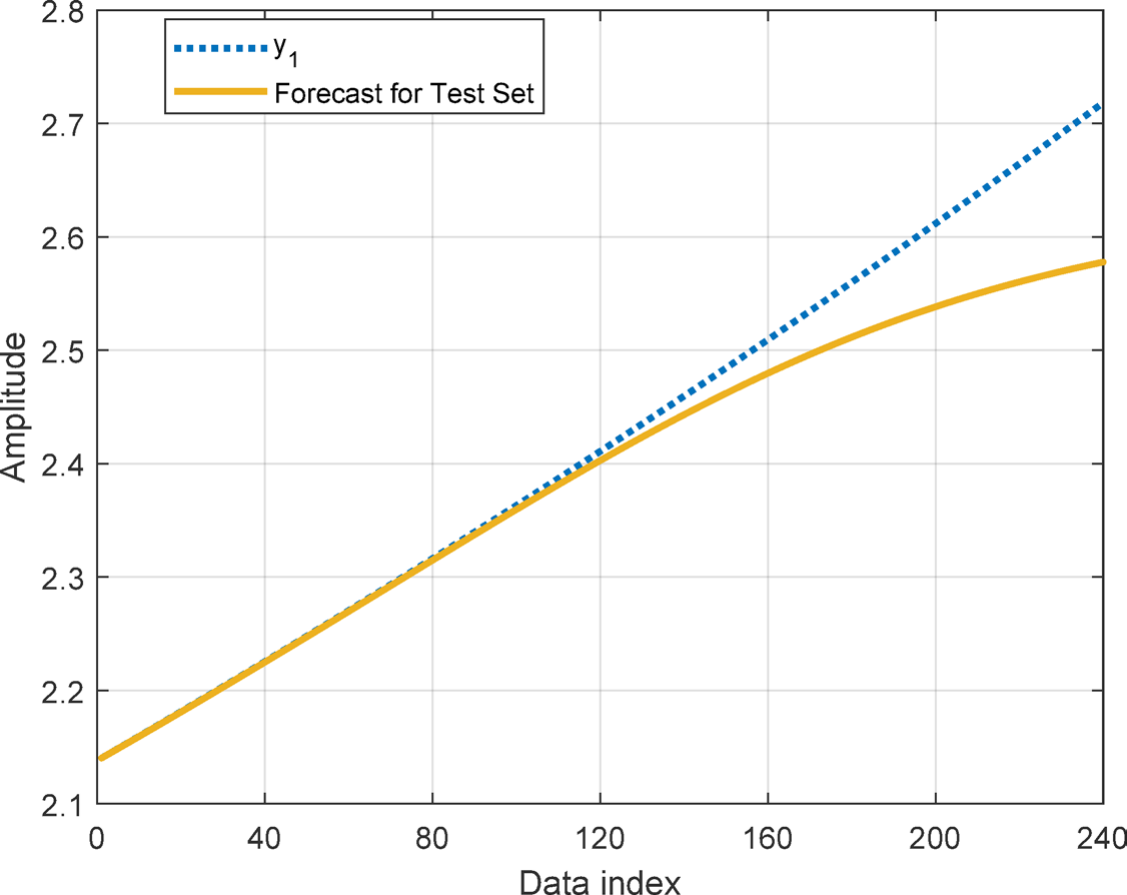
Forecast performance over test set for the benchmark function $\:{y}_{1}$.

**Fig. 7:**
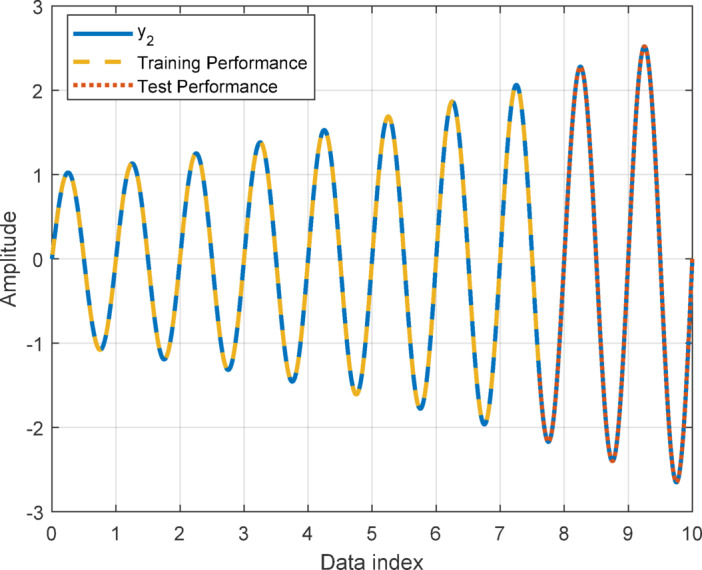
Training and test performances for the benchmark function $\:{y}_{2}$.

**Fig. 8:**
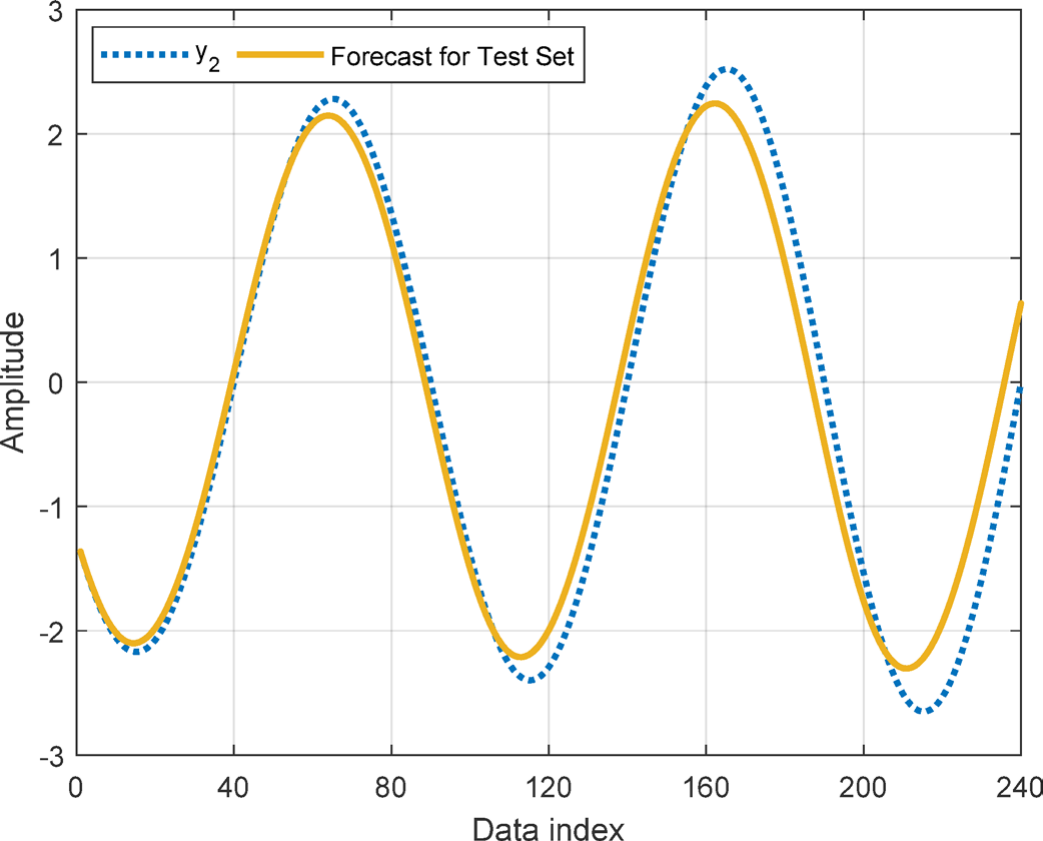
Forecast performance over test set for the benchmark function $\:{y}_{2}$.

**Fig. 9:**
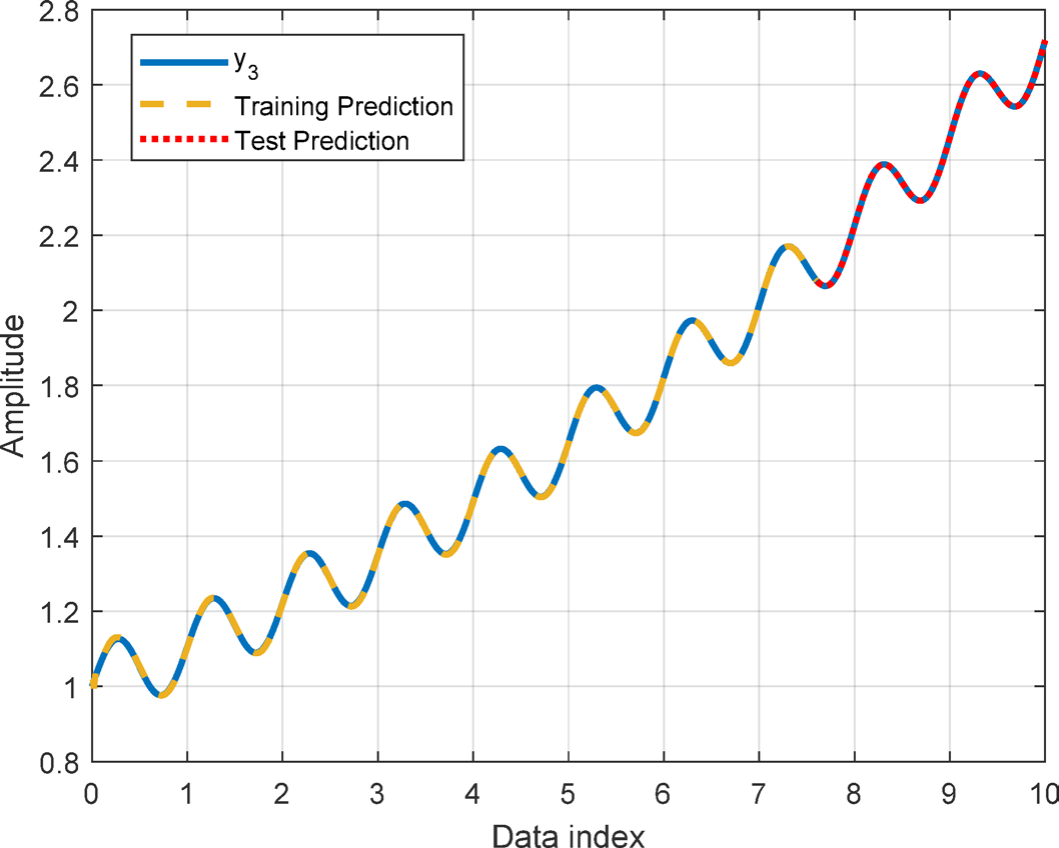
Training and test performance for the benchmark function $\:{y}_{3}$.

**Fig. 10:**
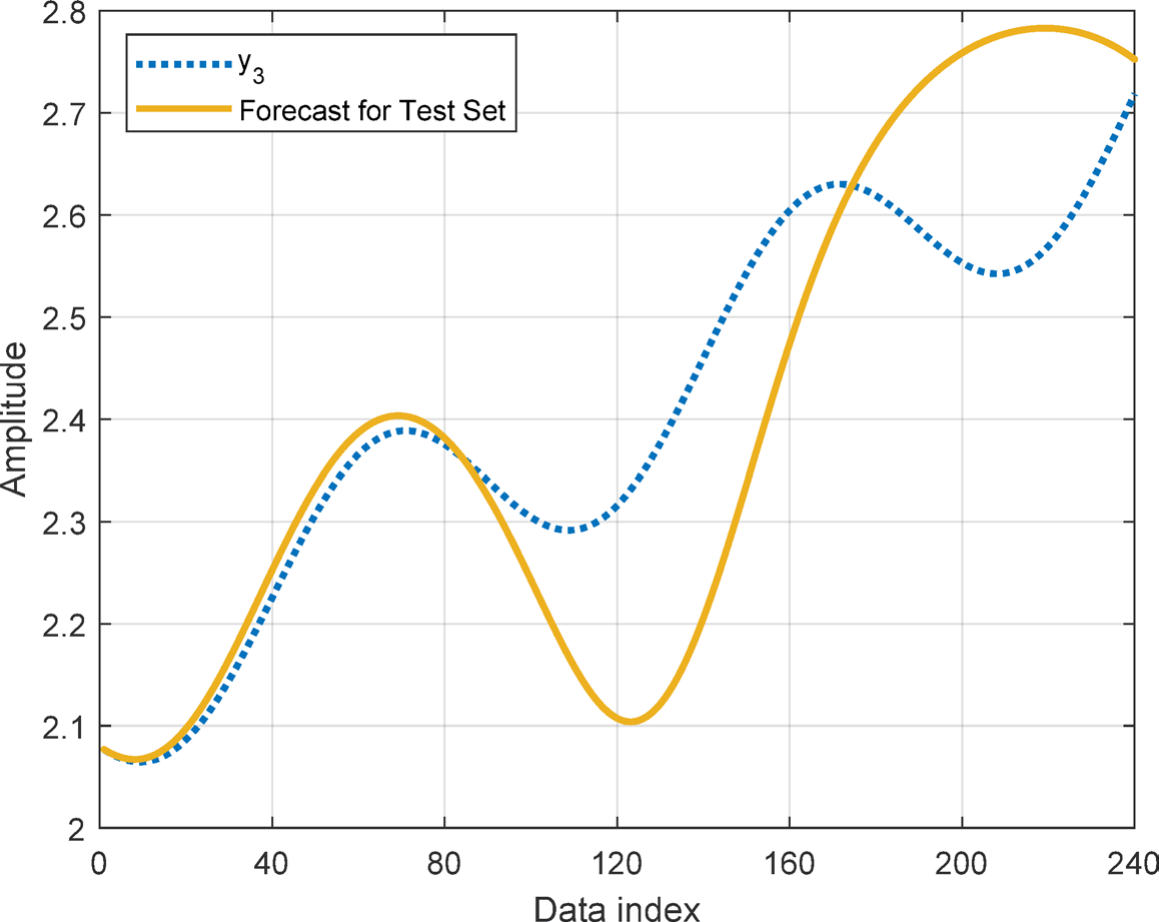
Forecast performance over test set for the benchmark function $\:{y}_{3}$.

**Fig. 11:**
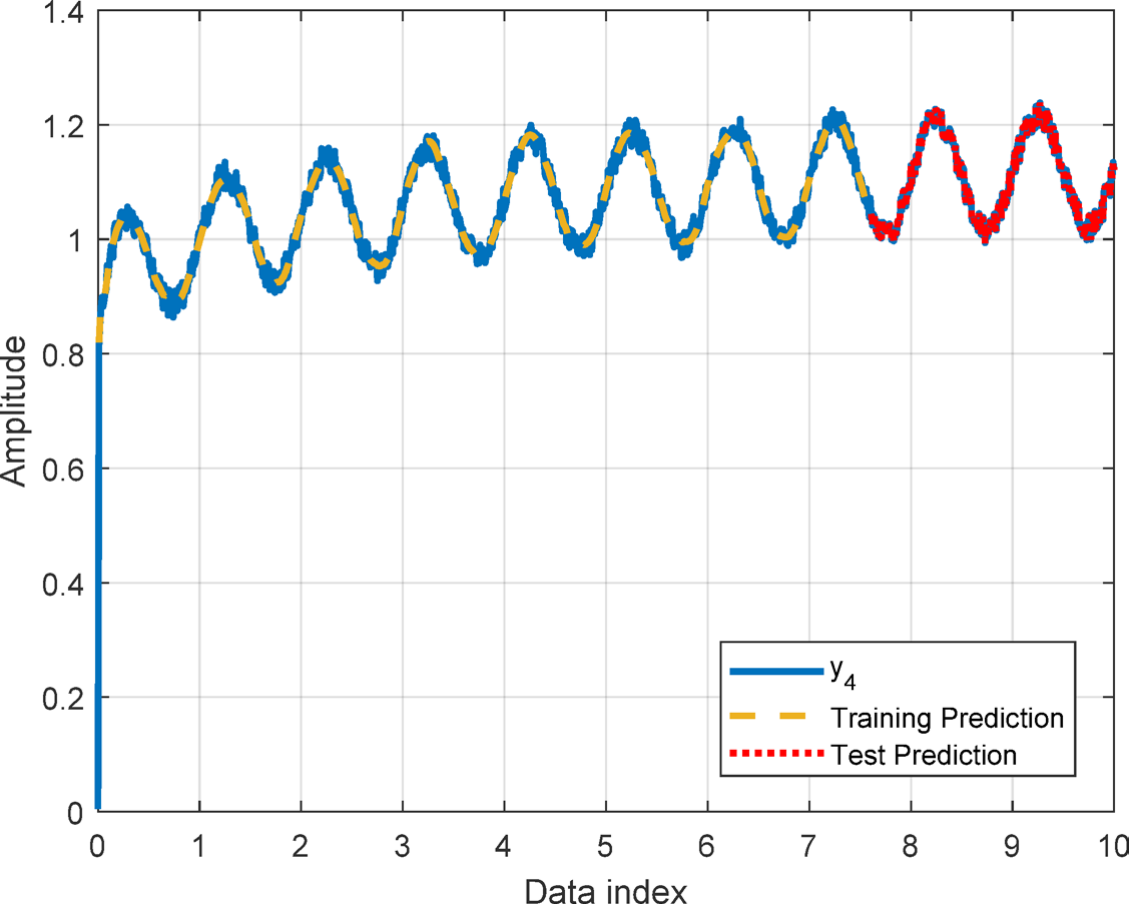
Training and test performance for the benchmark function $\:{y}_{4}$.

**Fig. 12:**
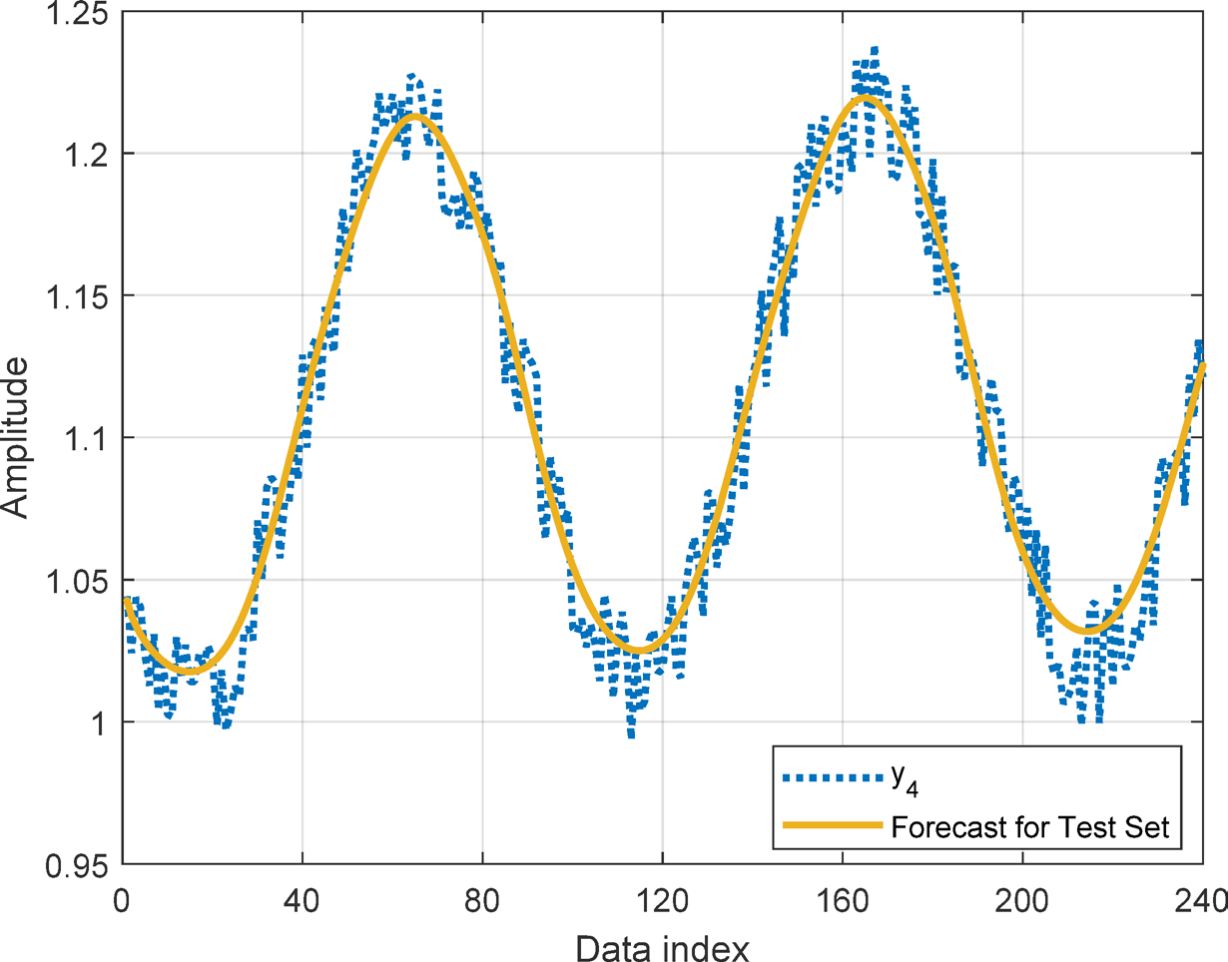
Forecast performance over test set for the benchmark function $\:{y}_{4}$.

In this section, to assess the performance of the proposed AT-LSTM model, the prediction performances on both the training and test sets for the benchmark functions were analyzed. Figures 5, 7, 9, and 11 show predictions of AT-LSTM models for training and test datasets for benchmark functions in Table 2. One can observe that the AT-LSTM model can accurately predict training and test data and provides satisfactory training and test performances. However, complication related to the long-term forecasting process, consequences of the error accumulation effect, is apparent in Figs. 6, 8, 10, and 12. These figures show the forecasting performances over the test dataset for the benchmark functions, and as forecasting horizon expands, divergences from test data becomes more apparent. Those divergences clearly indicate decreases in consistency of long-term forecasting and it better reveals long-term forecast performance of models. Therefore, the forecasting performances over test dataset should be considered in order to estimate practical performance of the forecaster models. Major reason of these growing errors is the accumulation of each prediction error in the feedback loop as illustrated in Fig. 3. In these figures, one observes that the forecast performance is acceptable up to about the first 80 data in the 240 data forecast horizon. Although the AT-LSTM model exhibits notable training and test performances in predicting the training and test sets, the long-term forecasting effort can maintain consistency up to a window of 80 data in forecast horizon for these benchmark functions. It should be noted that consideration of only training and test performances cannot sufficient to evaluate forecaster model performance.

### Comparison of the forecasting performance of several LSTM models

This section presents performance data for the AT-LSTM model and other LSTM networks from literature. The proposed AT-LSTM consists of two independently parameterized LSTM branches. Each branch contains an LSTM layer with 128 hidden units, and no weight sharing is employed. The outputs of the two branches are merged using an addition layer, after which the combined representation is passed to a two-stage fully connected decoder composed of a 10-unit fully connected layer followed by a 1-unit output layer. A standard regression layer is used for loss computation. No dropout layers or explicit L2 regularization are used; stabilization is primarily achieved through gradient thresholding and the learning-rate schedule (Detailed descriptions of the model architectures are provided in the Supplementary Material). The training, test, and forecast performance of these models allow us to discuss the suitability of this model for long-term forecasting of the global temperature anomaly. The parameters and their values ​​used in training are given in Table 3.

**Table 3:** Parameters used in training processes.

Parameter	Value/method
Epochs	500
Initial learning rate	0.005
Optimizer	Adaptive moment estimation (Adam)
Gradient threshold	1
Learning rate schedule	Piecewise
Learning rate drop period	125
Learning rate drop factor	0.2

- Training performance evaluation

Synthetic and real datasets were utilized for rigorous performance evaluation. For synthetic data generation, 1000 sequential sample ​​of each benchmark function were used. For real data, the five-year average temperature anomaly data of the Berkeley Temperature Anomaly dataset, consisting of 2038 data, were implemented. For all datasets, the last 240 data sample are used for the test set. The remaining part is allocated for the training set, and they are used for training of models. The training process was repeated 10 times for each model architecture, and the average values ​​of the training performance indices were calculated for both the benchmark functions ($\:{\mathrm{y}}_{1}$, $\:{y}_{2}$, $\:{y}_{3}$) and the temperature anomaly. Results are reported in Tables 4, 5 and 6. One observes that some models can provide better performances than the proposed AT-LSTM model. However, it should be noticed that the training performance does not truly express long-term forecasting performance of models. In fact, it can indicate the data reproduction performance of models over the training dataset.

**Table 4:** Average of root mean square error (RMSE) for training performance.

Architecture	$\:{\boldsymbol{y}}_{1}$	$\:{\boldsymbol{y}}_{2}$	$\:{\boldsymbol{y}}_{3}$	$\:{\boldsymbol{y}}_{4}$	Temp. Anomaly
AT-LSTM	0.000407	0.001535	0.000982	0.013799	0.004065
Multiplication of Twin LSTM	0.007753	0.004792	0.008237	0.013846	0.006370
Single LSTM with 128 hidden layers	0.001513	0.002058	0.002513	0.014294	0.005962
Single LSTM with 256 hidden layers	0.002019	0.001243	0.002894	0.014180	0.006430
Single biLSTM with 128 hidden layers	0.001678	0.006073	0.001855	0.000477	0.003867
Single biLSTM with 256 hidden layers	0.001949	0.006625	0.002014	0.000346	0.004030
Two LSTM (stacked and DC LSTM)^39^	0.000603	0.001666	0.001240	0.013723	0.006240
LSTM with 2-layer CNN in series^14^	0.000258	0.002615	0.001482	0.001808	0.002402
CNN-LSTM connected in parallel^19^	0.001250	0.004529	0.001987	0.009687	0.002835
GRU-LSTM connected in parallel^19^	0.000617	0.005142	0.003198	0.015100	0.003882
Attention-LSTM^40^	0.004725	0.005743	0.005856	0.015038	0.006966

**Table 5:** Average of mean absolute error (MAE) for training performance.

Architecture	$\:{\boldsymbol{y}}_{1}$	$\:{\boldsymbol{y}}_{2}$	$\:{\boldsymbol{y}}_{3}$	$\:{\boldsymbol{y}}_{4}$	Temp. Anomaly
AT-LSTM	0.000206	0.000764	0.000701	0.011760	0.003046
Multiplication of Twin LSTM	0.000634	0.001452	0.001002	0.011810	0.004227
Single LSTM with 128 hidden layers	0.000218	0.001316	0.000514	0.012254	0.004296
Single LSTM with 256 hidden layers	0.000214	0.000847	0.000549	0.012152	0.004548
Single biLSTM with 128 hidden layers	0.000416	0.001488	0.000520	0.000182	0.002569
Single biLSTM with 256 hidden layers	0.000476	0.001482	0.000545	0.000200	0.002544
Two LSTM (stacked and DC LSTM)^39^	0.000252	0.001099	0.000616	0.011718	0.004433
LSTM with 2-layer CNN in series^14^	0.000179	0.001981	0.001075	0.001252	0.001870
CNN-LSTM connected in parallel^19^	0.000263	0.001823	0.001103	0.007802	0.002122
GRU-LSTM connected in parallel^19^	0.000510	0.002197	0.002673	0.012771	0.002921
Attention-LSTM^40^	0.001257	0.003884	0.002626	0.012831	0.004969

**Table 6:** Average of R^2^-score for training performance.

Architecture	$\:{\boldsymbol{y}}_{1}$	$\:{\boldsymbol{y}}_{2}$	$\:{\boldsymbol{y}}_{3}$	$\:{\boldsymbol{y}}_{4}$	Temp. Anomaly
AT-LSTM	0.999998	0.999998	0.999991	0.972816	0.999750
Multiplication of Twin LSTM	0.999407	0.999978	0.999366	0.972608	0.999381
Single LSTM with 128 hidden layers	0.999974	0.999996	0.999935	0.970835	0.999461
Single LSTM with 256 hidden layers	0.999950	0.999999	0.999917	0.971296	0.999371
Single biLSTM with 128 hidden layers	0.999973	0.999967	0.999969	0.999965	0.999773
Single biLSTM with 256 hidden layers	0.999964	0.999960	0.999962	0.999982	0.999751
Two LSTM (stacked and DC LSTM)^39^	0.999993	0.999997	0.999985	0.973104	0.999404
LSTM with 2-layer CNN in series^14^	0.999999	0.999994	0.999977	0.999446	0.999912
CNN-LSTM connected in parallel^19^	0.999980	0.999982	0.999964	0.986590	0.999878
GRU-LSTM connected in parallel^19^	0.999996	0.999974	0.999906	0.967387	0.999771
Attention-LSTM^40^	0.999786	0.999970	0.999687	0.967709	0.999258

b) Test performance evaluation

High prediction performance for the training dataset does not ensure high performance for test dataset because test data were not used in the training process of the models. If the prediction performance severely decreases for unseen data in the test set, it is an indication of the overfitting case. The overfitted models are useless in terms of prediction practice because these models cannot learn general relations from data and they can exhibit very poor prediction performances for unseen data. Generalization, which implies learning general relations from data, can provides satisfactory prediction performance for unseen data. To detect overfitting state or evaluate generalization case, test datasets was allocated only for testing of the trained model for unseen data. In this study, the last 240 values ​​of the dataset were allocated for the test dataset. In our test, training of forecaster models was repeated 10 times and mean and standard deviation values ​​of the test performance indices were reported for both the benchmark function and the temperature anomaly in Tables 7, 8 and 9. When both the training performance (Tables 4, 5 and 6) and the test performance (Tables 7, 8 and 9) are considered together, one observes that the performance of the proposed AT-LSTM model indicate improved generalization performance because it exhibits better performances for the majority of test datasets. Particularly, the AT-LSTM model exhibits the highest R^2^-score (0.999427 for temperature anomaly dataset), the lowest RMSE value (0.003335 for temperature anomaly dataset) and the lowest MAE value (0.002484 for temperature anomaly dataset) than those of other models. These findings reveal that the AT-LSTM model has improved generalization for real dataset. This improvement can be closely related with the property that the AT-LSTM is capable of learning uncoupled dynamics in the temperature anomaly dataset.

**Table 7:** Root mean square error (RMSE) for test performance (mean ± std).

Architecture	$\:{\boldsymbol{y}}_{1}$	$\:{\boldsymbol{y}}_{2}$	$\:{\boldsymbol{y}}_{3}$	$\:{\boldsymbol{y}}_{4}$	Temp. Anomaly
AT-LSTM	0.0054 ± 0.004	0.1065 ± 0.000	0.0050 ± 0.001	0.0246 ± 0.007	0.0033 ± 0.001
Multiplication of Twin LSTM	0.1426 ± 0.071	0.1032 ± 0.002	0.0727 ± 0.099	0.0210 ± 0.006	0.0212 ± 0.014
Single LSTM with 128 hidden layers	0.0108 ± 0.005	0.1060 ± 0.001	0.0069 ± 0.001	0.0158 ± 0.001	0.0086 ± 0.003
Single LSTM with 256 hidden layers	0.0089 ± 0.007	0.1062 ± 0.001	0.0049 ± 0.000	0.0167 ± 0.002	0.0148 ± 0.008
Single biLSTM with 128 hidden layers	0.0373 ± 0.013	0.1041 ± 0.001	0.0169 ± 0.006	0.0211 ± 0.000	0.0200 ± 0.008
Single biLSTM with 256 hidden layers	0.0338 ± 0.010	0.1041 ± 0.001	0.0115 ± 0.003	0.0211 ± 0.000	0.0151 ± 0.014
Two LSTM (stacked and DC LSTM)^39^	0.0343 ± 0.018	0.1040 ± 0.001	0.0288 ± 0.015	0.0170 ± 0.001	0.0199 ± 0.009
LSTM with 2-layer CNN in series^14^	0.1589 ± 0.019	0.1251 ± 0.018	0.1092 ± 0.014	0.0243 ± 0.002	0.1160 ± 0.016
CNN-LSTM connected in parallel^19^	0.0132 ± 0.005	0.1060 ± 0.003	0.0090 ± 0.002	0.0197 ± 0.003	0.0113 ± 0.008
GRU-LSTM connected in parallel^19^	0.0259 ± 0.023	0.1040 ± 0.004	0.0131 ± 0.006	0.0151 ± 0.001	0.0144 ± 0.011
Attention-LSTM^40^	1.8913 ± 0.389	1.2599 ± 0.388	2.0774 ± 0.678	0.0707 ± 0.023	1.4983 ± 0.252

**Table 8:** Mean absolute error (MAE) for test performance (mean ± std).

Architecture	$\:{\boldsymbol{y}}_{1}$	$\:{\boldsymbol{y}}_{2}$	$\:{\boldsymbol{y}}_{3}$	$\:{\boldsymbol{y}}_{4}$	Temp. Anomaly
AT-LSTM	0.0041 ± 0.003	0.0942 ± 0.001	0.0043 ± 0.000	0.0199 ± 0.005	0.0025 ± 0.000
Multiplication of Twin LSTM	0.1107 ± 0.061	0.0898 ± 0.002	0.0506 ± 0.060	0.0173 ± 0.005	0.0148 ± 0.010
Single LSTM with 128 hidden layers	0.0076 ± 0.003	0.0933 ± 0.001	0.0058 ± 0.001	0.0133 ± 0.001	0.0065 ± 0.002
Single LSTM with 256 hidden layers	0.0064 ± 0.005	0.0938 ± 0.001	0.0042 ± 0.000	0.0140 ± 0.002	0.0112 ± 0.006
Single biLSTM with 128 hidden layers	0.0276 ± 0.010	0.0921 ± 0.001	0.0105 ± 0.004	0.0171 ± 0.000	0.0164 ± 0.008
Single biLSTM with 256 hidden layers	0.0257 ± 0.008	0.0923 ± 0.001	0.0069 ± 0.003	0.0171 ± 0.000	0.0103 ± 0.015
Two LSTM (stacked and DC LSTM)^39^	0.0242 ± 0.013	0.0921 ± 0.001	0.0226 ± 0.012	0.0144 ± 0.001	0.0144 ± 0.006
LSTM with 2-layer CNN in series^14^	0.1215 ± 0.017	0.1037 ± 0.009	0.0691 ± 0.010	0.0185 ± 0.001	0.0753 ± 0.012
CNN-LSTM connected in parallel^19^	0.0058 ± 0.003	0.0941 ± 0.002	0.0053 ± 0.001	0.0156 ± 0.002	0.0043 ± 0.001
GRU-LSTM connected in parallel^19^	0.0196 ± 0.018	0.1040 ± 0.004	0.0107 ± 0.005	0.0127 ± 0.001	0.0105 ± 0.008
Attention-LSTM^40^	1.8837 ± 0.391	1.1463 ± 0.353	2.0692 ± 0.680	0.0623 ± 0.022	1.4915 ± 0.253

**Table 9:** R^2^-score for test performance (mean ± std).

Architecture	$\:{\boldsymbol{y}}_{1}$	$\:{\boldsymbol{y}}_{2}$	$\:{\boldsymbol{y}}_{3}$	$\:{\boldsymbol{y}}_{4}$	Temp. Anomaly
AT-LSTM	0.9985 ± 0.002	0.9961 ± 0.000	0.9992 ± 0.000	0.9577 ± 0.065	0.9994 ± 0.000
Multiplication of Twin LSTM	0.1142 ± 0.803	0.9963 ± 0.000	0.5625 ± 1.191	0.9088 ± 0.062	0.9685 ± 0.050
Single LSTM with 128 hidden layers	0.9951 ± 0.004	0.9961 ± 0.000	0.9985 ± 0.001	0.9521 ± 0.005	0.9960 ± 0.002
Single LSTM with 256 hidden layers	0.9958 ± 0.005	0.9961 ± 0.000	0.9993 ± 0.000	0.9461 ± 0.015	0.9861 ± 0.017
Single biLSTM with 128 hidden layers	0.9453 ± 0.032	0.9963 ± 0.000	0.9902 ± 0.006	0.9150 ± 0.000	0.9774 ± 0.016
Single biLSTM with 256 hidden layers	0.9564 ± 0.022	0.9963 ± 0.000	0.9956 ± 0.003	0.9150 ± 0.000	0.9795 ± 0.046
Two LSTM (stacked and DC LSTM)^39^	0.9475 ± 0.053	0.9963 ± 0.000	0.9678 ± 0.033	0.9445 ± 0.008	0.9764 ± 0.025
LSTM with 2-layer CNN in series^14^	0.0880 ± 0.210	0.9945 ± 0.002	0.6251 ± 0.092	0.8874 ± 0.018	0.3141 ± 0.185
CNN-LSTM connected in parallel^19^	0.9930 ± 0.005	0.9961 ± 0.000	0.9973 ± 0.001	0.9246 ± 0.026	0.9908 ± 0.015
GRU-LSTM connected in parallel^19^	0.9590 ± 0.083	0.9963 ± 0.000	0.9938 ± 0.005	0.9566 ± 0.006	0.9840 ± 0.026
Attention-LSTM^40^	-131.45 ± 55.10	0.4094 ± 0.333	-145.52 ± 97.6	-0.045 ± 0.586	-114.28 ± 36.77

c) Evaluation of forecast performances on test dataset

For estimation of future forecast performance of models, forecasting tests were carried out in test dataset. Here, the first data of test dataset is applied to the models and predictions of models are used to feed inputs of models in order to forecast the next data. This feedback loop is performed 240 times to perform long-term forecasting trough test dataset. For 10 repeated modeling of each model architecture is carried out and the mean and standard deviation values ​​of the forecast performances were calculated for both the benchmark functions and the temperature anomaly in Tables 10, 11 and 12. These performance data were considered, the AT-LSTM model mostly exhibited better forecasting performance on the test dataset. It provided the highest R^2^-score (0.521012 for temperature anomaly dataset), the lowest RMSE value (0.093977 for temperature anomaly dataset) and the lowest MAE value (0.072846 for temperature anomaly dataset). This performance improvement can indicate advantages of the AT-LSTM model, which is the learning uncoupled dynamics in the temperature anomaly dataset.

**Table 10:** Root mean square error (RMSE) for forecast performance (mean ± std).

Architecture	$\:{\boldsymbol{y}}_{1}$	$\:{\boldsymbol{y}}_{2}$	$\:{\boldsymbol{y}}_{3}$	$\:{\boldsymbol{y}}_{4}$	Temp. Anomaly
AT-LSTM	0.0714 ± 0.015	0.4264 ± 0.084	0.3838 ± 0.102	0.0211 ± 0.004	0.0940 ± 0.029
Multiplication of Twin LSTM	0.2514 ± 0.034	0.2857 ± 0.068	0.5425 ± 0.228	0.0227 ± 0.004	0.2313 ± 0.040
Single LSTM with 128 hidden layers	0.1994 ± 0.026	0.3747 ± 0.105	0.5340 ± 0.472	0.0251 ± 0.009	0.1946 ± 0.054
Single LSTM with 256 hidden layers	0.0893 ± 0.054	0.3733 ± 0.080	0.3771 ± 0.094	0.0222 ± 0.007	0.3004 ± 0.131
Single biLSTM with 128 hidden layers	0.5214 ± 0.036	1.7388 ± 0.015	0.5301 ± 0.031	0.0824 ± 0.001	0.7754 ± 0.031
Single biLSTM with 256 hidden layers	0.5227 ± 0.060	1.7532 ± 0.024	0.5344 ± 0.056	0.0816 ± 0.001	0.7909 ± 0.050
Two LSTM (stacked and DC LSTM)^39^	0.2003 ± 0.030	0.3271 ± 0.096	0.3348 ± 0.026	0.0223 ± 0.002	0.2372 ± 0.061
LSTM with 2-layer CNN in series^14^	0.9255 ± 0.004	1.7259 ± 0.017	0.9165 ± 0.007	0.0866 ± 0.002	0.9037 ± 0.022
CNN-LSTM connected in parallel^19^	1.2081 ± 0.949	1.7178 ± 0.058	1.5010 ± 1.230	0.0912 ± 0.002	1.4521 ± 1.031
GRU-LSTM connected in parallel^19^	0.1382 ± 0.060	0.5442 ± 0.312	0.4675 ± 0.112	0.0678 ± 0.034	0.2853 ± 0.140
Attention-LSTM^40^	0.1767 ± 0.048	1.7541 ± 0.013	0.9578 ± 0.124	0.0895 ± 0.007	0.9213 ± 0.059

**Table 11:** Mean absolute error (MAE) for forecast performance (mean ± std).

Architecture	$\:{\boldsymbol{y}}_{1}$	$\:{\boldsymbol{y}}_{2}$	$\:{\boldsymbol{y}}_{3}$	$\:{\boldsymbol{y}}_{4}$	Temp. Anomaly
AT-LSTM	0.0492 ± 0.012	0.3295 ± 0.064	0.3398 ± 0.099	0.0172 ± 0.003	0.0728 ± 0.019
Multiplication of Twin LSTM	0.2028 ± 0.034	0.2278 ± 0.057	0.4650 ± 0.185	0.0184 ± 0.003	0.1889 ± 0.041
Single LSTM with 128 hidden layers	0.1553 ± 0.024	0.2970 ± 0.081	0.4133 ± 0.395	0.0202 ± 0.007	0.1562 ± 0.051
Single LSTM with 256 hidden layers	0.0653 ± 0.041	0.2936 ± 0.063	0.3224 ± 0.098	0.0180 ± 0.005	0.2559 ± 0.121
Single biLSTM with 128 hidden layers	0.4772 ± 0.035	1.4964 ± 0.008	0.4967 ± 0.032	0.0672 ± 0.000	0.7567 ± 0.032
Single biLSTM with 256 hidden layers	0.4818 ± 0.058	1.5064 ± 0.004	0.5012 ± 0.059	0.0668 ± 0.000	0.7729 ± 0.051
Two LSTM (stacked and DC LSTM)^39^	0.1564 ± 0.028	0.2593 ± 0.078	0.2865 ± 0.025	0.0185 ± 0.002	0.1918 ± 0.064
LSTM with 2-layer CNN in series^14^	0.9075 ± 0.004	1.5546 ± 0.019	0.8958 ± 0.008	0.0696 ± 0.001	0.8911 ± 0.023
CNN-LSTM connected in parallel^19^	1.1787 ± 0.942	1.5354 ± 0.072	1.4530 ± 1.187	0.0723 ± 0.002	1.4284 ± 1.018
GRU-LSTM connected in parallel^19^	0.1116 ± 0.050	0.4403 ± 0.255	0.4285 ± 0.116	0.0553 ± 0.026	0.2391 ± 0.134
Attention-LSTM^40^	0.1358 ± 0.041	1.5802 ± 0.011	0.9393 ± 0.128	0.0718 ± 0.005	0.9093 ± 0.060

It is noteworthy to consider performance data in Table 11. The table reveals that future forecast error of the AT-LSTM model has an average 0.072846 °C for 10 repeated modelling. This forecast error level in test set implies that forecasting error for the following 240 months in future can be expected at the level of 0.072846 °C or slightly more. In other words, for the 240 months future temperature forecasts, the nearest error level expectation of AT-LSTM model can be estimated as 0.072846 °C. This error level for long-term global temperature anomaly forecasting is meaningful for climate studies. For instance, for the model CNN-LSTM connected in parallel, an average error of 1.428369 °C was observed and it expresses the nearest error level expectation for 240 months future forecasting of this model. When compared, 240 months forecast error expectation level of the AT-LSTM model is much less (about 5%) and therefore the temperature anomaly forecasting of AT-LSTM model can be more consistent in average.

**Table 12:** R^2^-score for forecast performance (mean ± std).

Architecture	$\:{\boldsymbol{y}}_{1}$	$\:{\boldsymbol{y}}_{2}$	$\:{\boldsymbol{y}}_{3}$	$\:{\boldsymbol{y}}_{4}$	Temp. Anomaly
AT-LSTM	0.8109 ± 0.081	0.9355 ± 0.026	-3.8574 ± 2.05	0.9129 ± 0.030	0.5210 ± 0.259
Multiplication of Twin LSTM	-1.2909 ± 0.687	0.9706 ± 0.014	-9.567 ± 9.716	0.8997 ± 0.033	-1.7515 ± 0.965
Single LSTM with 128 hidden layers	-0.4394 ± 0.359	0.9485 ± 0.033	-14.04 ± 31.47	0.8666 ± 0.092	-1.0294 ± 1.097
Single LSTM with 256 hidden layers	0.6218 ± 0.331	0.9502 ± 0.021	-3.651 ± 2.165	0.8976 ± 0.079	-4.2889 ± 4.255
Single biLSTM with 128 hidden layers	-8.7414 ± 1.339	-0.0366 ± 0.02	-7.734 ± 1.027	-0.2937 ± 0.020	-29.1605 ± 2.45
Single biLSTM with 256 hidden layers	-8.8616 ± 2.303	-0.054 ± 0.029	-7.938 ± 1.871	-0.2681 ± 0.028	-30.445 ± 4.04
Two LSTM (stacked and DC LSTM)^39^	-0.4604 ± 0.446	0.9605 ± 0.025	-2.493 ± 0.557	0.9004 ± 0.019	-1.9867 ± 1.546
LSTM with 2-layer CNN in series^14^	-29.553 ± 0.259	-0.0214 ± 0.02	-25.03 ± 0.391	-0.4307 ± 0.074	-39.9226 ± 1.95
CNN-LSTM connected in parallel^19^	-79.983 ± 153.5	-0.0127 ± 0.06	-111.0 ± 203.4	-0.5854 ± 0.069	-152.53 ± 224.1
GRU-LSTM connected in parallel^19^	0.2044 ± 0.582	0.8685 ± 0.143	-6.122 ± 3.334	-0.0707 ± 1.085	-3.9579 ± 4.72
Attention-LSTM^40^	-0.1866 ± 0.518	-0.0549 ± 0.01	-27.856 ± 6.18	-0.5338 ± 0.24	-41.6746 ± 5.11

**Fig. 13:**
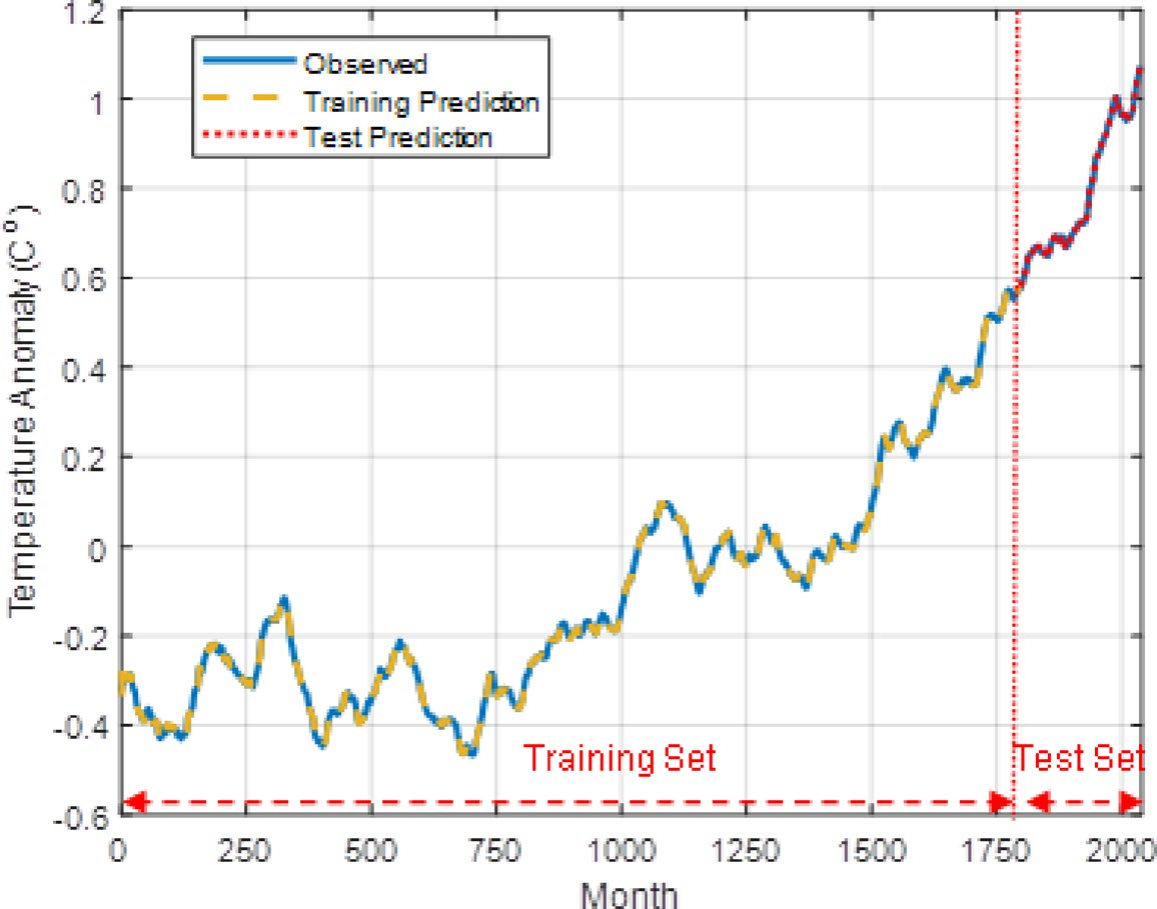
The temperature predictions of 10th LSTM model for training and test set.

**Fig. 14:**
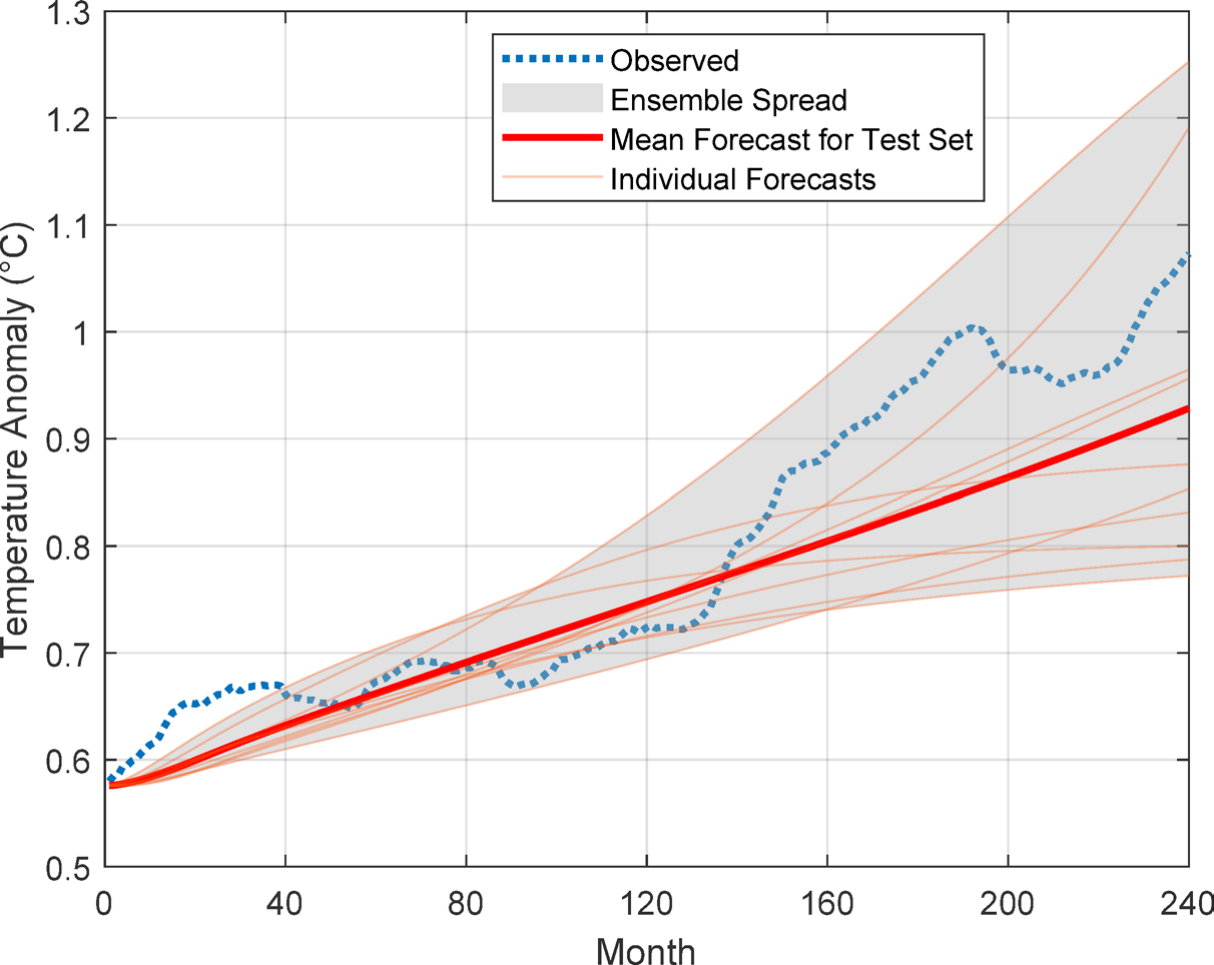
Forecast performance over test set of temperature dataset.

Figure 13 shows the temperature prediction of 10th AT-LSTM model for the training set and test set allocations throughout the Berkeley temperature anomaly dataset. The prediction on training and test sets are very consistent with the observed data and indicates high prediction performances. However, the long-term forecast performance over test set should be considered to estimate the practical forecast performance. The temperature anomaly forecasting over the test set for 10 AT-LSTM models and their average forecasting is illustrated in Fig. 14. This figure reveals that forecasts of AT-LSTM models are consistent with the temperature anomaly trend of 240 months test data to some extent. The average forecasting of 10 AT-LSTM models is rather consistent with trend of the test data. However, the average forecasts are enough consistent with the first 140 months data and almost express the trend curve of the 5 year-averaged 140 months global temperature anomaly data. As expected, although next data prediction performances on training and test dataset are very accurate as illustrated in Fig. 13, the long-term forecasting on test dataset is not very accurate as illustrated in Fig. 14. The main reason for effect is the long-term forecast error accumulation in the long-term forecasting process as explained in A forecast performance evaluation procedure for time series data.

d) Future projection for global temperature anomaly trends based on forecasts of AT-LSTM models

**Fig. 15:**
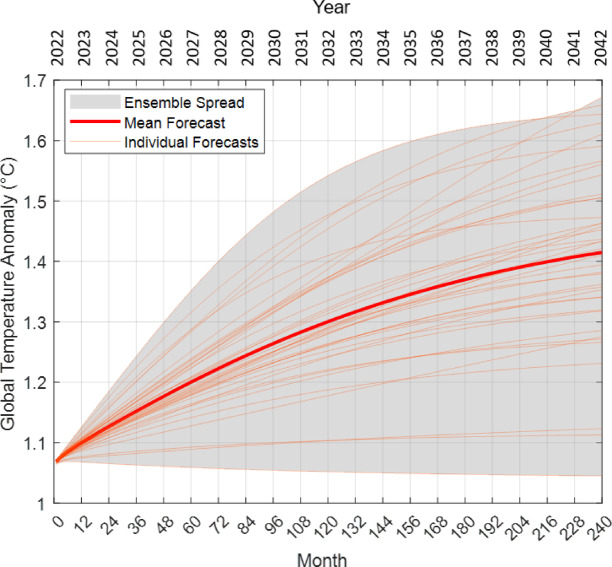
Future forecasts of global temperature anomaly by AT-LSTM models and the average forecast line (red line) for next 20 years.

This section discusses global temperature anomaly forecasts of AT-LSTM models for the next 20 years (240 months) from 2022 to 2042. Figure 15 shows the temperature anomaly forecasts of 40 AT-LSTM models for the next 20 years, and average forecasts of these models were indicated with red solid line in the figure. To increase consistency of average forecasts, we trained 40 AT-LSTM models and show their forecasts with yellow thin lines. Based on the forecasting performance on test set in Table 11, the average forecast error can be expected at a level of 0.072846 °C. This error level assumes an ± 0.073 °C uncertainty range in in average forecasting error and indicates reliability of the average model.

Figure 15 demonstrates that all models forecast an increasing trend in the global temperature anomaly in next 20 years. The temperature anomaly forecast for the year 2042 is varied between 1.05 °C and 1.67 °C. The average of temperature anomaly forecasts (red line) for the year 2042 forecasts 1.415 °C ± 0.073 °C global temperature anomaly. The average temperature anomaly forecast of 1.415 °C by the year 2042 aligns with global temperature projections in the literature, confirming that the model provides reasonable and consistent forecasts with other studies. For example, the Intergovernmental Panel on Climate Change (IPCC) Sixth Assessment Report (2021)^41^ highlights that global temperatures could rise by 1.5 °C above pre-industrial levels by 2030–2050 under high-emission scenarios. The 1.415 °C forecasting by the 2042 from the AT-LSTM model is consistent with outputs from traditional climate models (e.g., CMIP6 models^42^ and emphasizes the growing confidence in machine learning-based approaches for climate forecasting. If the global temperature anomaly reaches at a level of 1.40 °C by 2040, climatic analyses for such a high temperature anomaly suggests that there will be urgent need for applying required environmental policies (The Paris Agreement (2015) and others) and feasible technological solutions^43–45^ in order to mitigate the global warming. The Paris Agreement (2015) aims to limit global warming to well below 2 °C, with efforts to limit it to 1.5 °C. The AT-LSTM model’s forecast reveals potential of the current trend coming near or exceeding the 1.5 °C threshold by mid-century. This analysis highlights the urgency of implementing climate mitigation measures to reduce greenhouse gas emissions and prevent catastrophic climate impacts, as emphasized in reports by organizations like the IPCC and the World Meteorological Organization (WMO).

The forecasts for 2042, which ranges between 1.05 °C and 1.67 °C, reflect the training diversity of models, which can be clearly observed in the long-term climate forecasts. However, all trained models suggested the potential of increasing trend in the temperature anomaly. As noted in the literature, Tebaldi et al. (2021)^42^ emphasized that future temperature forecasts were subject to significant uncertainty due to numerous unpredictable factors such as future of greenhouse gas emissions, dynamics and chaotic response of climate systems, natural climate variabilities, world policy, and socio-economic developments, several other anthropogenic factors. The current forecasts are based on collected data in a period of (1850–2024) and the trained models assume that similar dynamics and factors continue in future. That is why, the model diversity and multi-model analyses is useful to consider possible scenarios in the forecasting and achieve the most probable estimates. Obviously, the diversity in model forecast and the uncertainty level should increase as the forecast horizon extends into the future.

## Discussion and conclusions

Data-driven modeling of climate responses and time series forecasting are very important in these days because Earth climate is very near to or exceeding a critical point, where monitoring current trends and estimation of future trends in the global warming problem is highly vital to maintain Earth habitability and future of humankind. Unfortunately, observations and measured climate data is quite limited to accurately model highly complex earth climate system. In this case, data-driven modeling and machine learning techniques can helpful to improve forecast model.

Training and performance evaluation forecaster model, which learn the dominant dynamics of the climate responses from insufficient sequential data, is a particularly challenging problem. To improve time series forecasting performance, three stages gain more importance; the first one is suitably preprocessing of raw data sequences (selection of sampling period, reducing noise and uncertainty in raw data etc.), the second one is the design of suitable models (models should be capable of exploring and learning responses of dominating dynamics (factors) from sequential data) and the third one is the dependable performance evaluation strategies (considering prediction performance in training and test dataset cannot reveal long-term future forecast performance of these models.) Some remarks of this study are summarized:

* An AT-LSTM model is proposed to learn two uncoupled dominating dynamic responses from the monthly temperature anomaly data. It involves two LSTMs (to learn response of two dominating dynamics from data sequence) and the neural decoder (to learn characteristics of measurement process).
* Benchmark functions were designed to establish a sterile and controlled test environment (without noise, uncertainty and disturbances) that resembles specific global temperature anomaly trends. The benchmark function is useful to reveal expressive capability of models for specific characteristics in applications.
* In order to reduce negative impacts of noise on the modeling process, the five-years averaged monthly sampled temperature anomaly data were used. The averaged data better expresses consistent trends in temperature anomaly observation and facilitates learning these trends by the forecaster models.
* The forecast performance on the test set is used to estimate long-term future forecast performance of models. It was emphasized that data prediction in training and test sets does not express long-term future forecast performance of the forecaster models.

The average temperature anomaly forecast of the AT-LSTM models was 1.415 °C with ± 0.073 °C average error expectation for a projection to the year 2042. It is consistent with the global temperature anomaly projections in the literature. Potential of rising temperature anomaly up to 1.415 °C in 20 years is very important because it is alerting point for urgently activating reduction policies and necessary climate mitigation measures. We highlight the urgency of implementing climate mitigation measures for reducing greenhouse gas emissions and other feasible solution to prevent catastrophic climate changes, as emphasized in reports by other organizations.

## Supplementary Information

Below is the link to the electronic supplementary material.

Supplementary Material 1

## Data Availability

The datasets used and/or analyzed during the current study are available from the corresponding author on reasonable request.
