# Supplementary material for: Global temperature anomaly prediction by using additive twin LSTM networks
Source: Sci Rep. 2026 Jan 28;16:6456. doi: 10.1038/s41598-026-37255-x (PMC12909854; doi:10.1038/s41598-026-37255-x)
Supplement: Supplementary file 1 — Supplementary Material 1 [file 41598_2026_37255_MOESM1_ESM.docx]

**Supplementary Material**

**Model Architectures**

The architectures of the models used in this study are given below:

Multiplicative twin LSTM model (MT-LSTM)


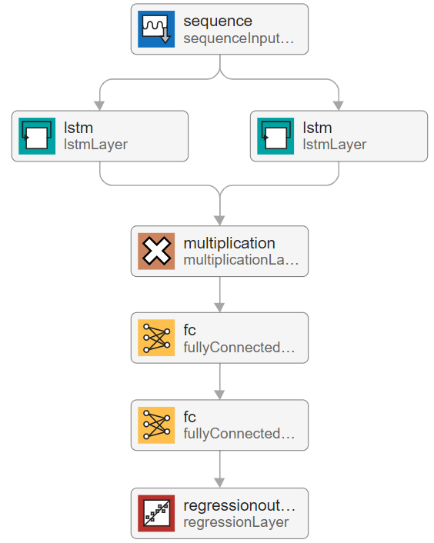


Additive twin LSTM model (AT-LSTM)


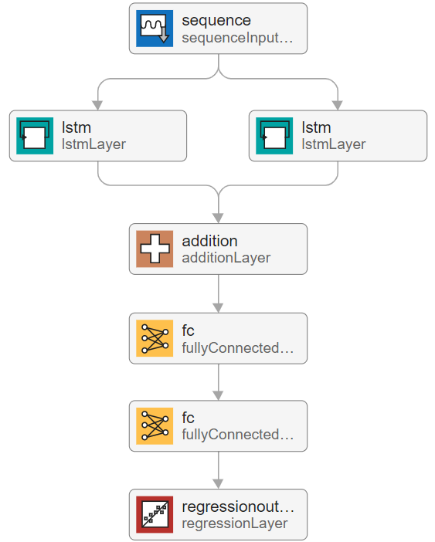


Attention-LSTM model^40^


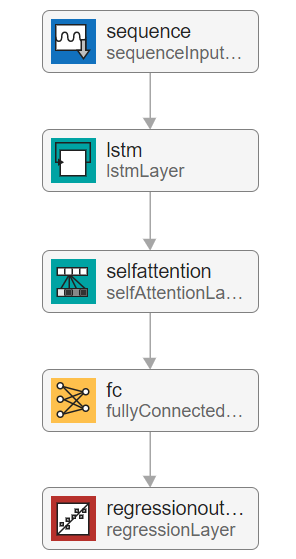


Single LSTM model


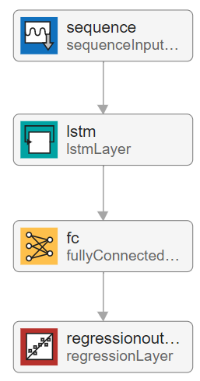


Single biLSTM model


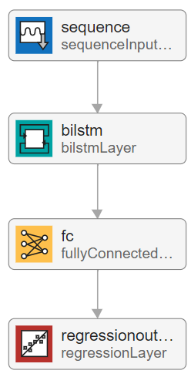


Two LSTM (Stacked and DC LSTM) model^39^


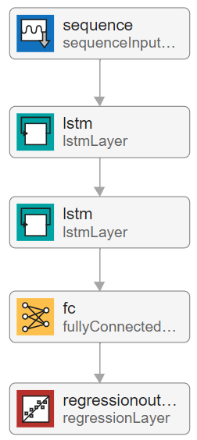


LSTM with 2-layer CNN in series model^14^


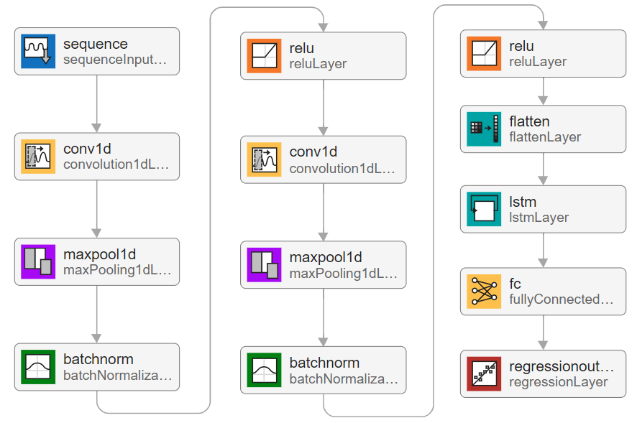


CNN-LSTM connected in parallel model^19^


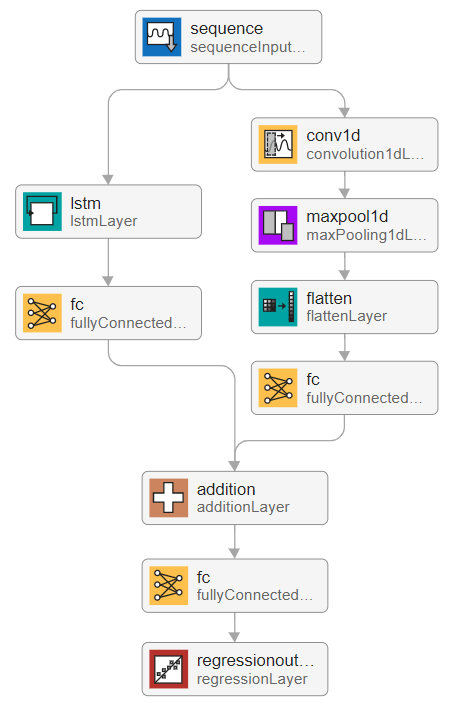


GRU-LSTM connected in parallel model^19^


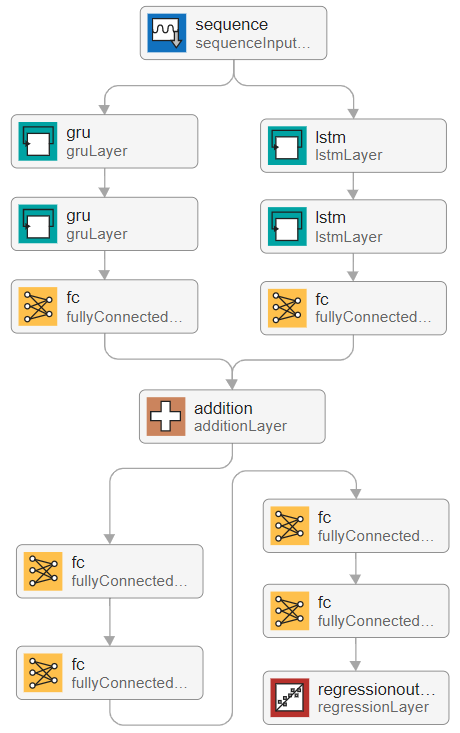


**Property** **(Multi-branch approximation to independent recurrent relations):** Let assume a composite recurrent relation that can be decomposed into $n$ number of the independent recurrent relation $x_{t+1}=\sum_{i=1}^{n} \gamma_{i}d_{t+1,i}$. For a long-term approximation ($t\in[0,\infty$)) with a random error $\varepsilon$, $n$ number of recurrent neural networks can approximate this composite recurrent relation in training state with decreasing loss function.

*Proof:*

Let’s assume that for a long-term approximation ($t\in[0,\infty$)), a recurrent neural network can approximate to an independent relation

${d_{t+1,i}=F}_{i}\left( d_{t,i},x_{t} \right)\pm\epsilon_{i}$ (A1)

If $n$ number of recurrent neural networks is combined in summing form, one obtains

$x_{t+1}=\sum_{i=1}^{n} \gamma_{i}F_{i}\left( d_{t,i},x_{t} \right)$ (A2)

It can be reorganized as

$x_{t+1}=\sum_{i=1}^{n} \gamma_{i}(d_{t+1,i}\pm\epsilon_{i})$ (A3)

Then,

$x_{t+1}=\sum_{i=1}^{n} \gamma_{i}d_{t+1,i}\pm\sum_{i=1}^{n} {\gamma_{i}\epsilon}_{i}$ (A4)

The first term is the learned independent recurrent relations by the combined recurrent neural networks. The second term $\pm\sum_{i=1}^{n} {\gamma_{i}\epsilon}_{i}$ expresses the random measurement noise that appears in the learning process as $\varepsilon=\pm\sum_{i=1}^{n} {\gamma_{i}\epsilon}_{i}$ . Consequently, all independent recurrent relations in sequence can be completely approximated by $n$ number of recurrent neural networks in summing form during training state. To minimize the loss function during the weight optimization, each recurrent neural network must approximate to an independent recurrent relation. Otherwise, the loss function does not decrease and training performance cannot be improved further. Hence, in case of two independent recurrent relations (dynamics), each LSTM branch should focus on one uncoupled recurrent relation to minimize the loss function. This inherent mathematical constraint ensures that the AT-LSTM model learns the most dominating two uncoupled recurrent relations in order to improve performance.
